# Quantification of pulmonary edema using automated lung segmentation on computed tomography in mechanically ventilated patients with acute respiratory distress syndrome

**DOI:** 10.1186/s40635-024-00685-w

**Published:** 2024-11-02

**Authors:** Alice Marguerite Conrad, Julia Zimmermann, David Mohr, Matthias F. Froelich, Alexander Hertel, Nils Rathmann, Christoph Boesing, Manfred Thiel, Stefan O. Schoenberg, Joerg Krebs, Thomas Luecke, Patricia R. M. Rocco, Matthias Otto

**Affiliations:** 1 Department of Anesthesiology and Critical Care Medicine, Faculty of Medicine, University Hospital Mannheim, University of Heidelberg, Theodor-Kutzer Ufer 1-3, 68165 Mannheim, Germany; 2 Department of Clinical Radiology and Nuclear Medicine, Faculty of Medicine, University Hospital Mannheim, University of Heidelberg, Theodor-Kutzer Ufer 1-3, 68165 Mannheim, Germany; 3 Laboratory of Pulmonary Investigation, Centro de Ciências da Saúde, Carlos Chagas Filho Institute of Biophysics, Federal University of Rio de Janeiro, Avenida Carlos Chagas Filho, 373, Bloco G-014, Ilha Do Fundão, Rio de Janeiro, Brazil

**Keywords:** ARDS, Pulmonary edema, Transpulmonary thermodilution, Extravascular lung water, Automated lung segmentation, Computed tomography

## Abstract

**Background:**

Quantification of pulmonary edema in patients with acute respiratory distress syndrome (ARDS) by chest computed tomography (CT) scan has not been validated in routine diagnostics due to its complexity and time-consuming nature. Therefore, the single-indicator transpulmonary thermodilution (TPTD) technique to measure extravascular lung water (EVLW) has been used in the clinical setting. Advances in artificial intelligence (AI) have now enabled CT images of inhomogeneous lungs to be segmented automatically by an intensive care physician with no prior radiology training within a relatively short time. Nevertheless, there is a paucity of data validating the quantification of pulmonary edema using automated lung segmentation on CT compared with TPTD.

**Methods:**

A retrospective study (January 2016 to December 2021) analyzed patients with ARDS, admitted to the intensive care unit of the Department of Anesthesiology and Critical Care Medicine, University Hospital Mannheim, who underwent a chest CT scan and hemodynamic monitoring using TPTD at the same time. Pulmonary edema was estimated using manually and automated lung segmentation on CT and then compared to the pulmonary edema calculated from EVLW determined using TPTD.

**Results:**

145 comparative measurements of pulmonary edema with TPTD and CT were included in the study. Estimating pulmonary edema using either automated lung segmentation on CT or TPTD showed a low bias overall (− 104 ml) but wide levels of agreement (upper: 936 ml, lower: − 1144 ml). In 13% of the analyzed CT scans, the agreement between the segmentation of the AI algorithm and a dedicated investigator was poor. Manual segmentation and automated segmentation adjusted for contrast agent did not improve the agreement levels.

**Conclusions:**

Automated lung segmentation on CT can be considered an unbiased but imprecise measurement of pulmonary edema in mechanically ventilated patients with ARDS.

**Supplementary Information:**

The online version contains supplementary material available at 10.1186/s40635-024-00685-w.

## Background

Acute respiratory distress syndrome (ARDS) is a life-threatening organ dysfunction with high morbidity and mortality characterized by the formation of pulmonary edema and ventilation–perfusion mismatch [1, 2]. Pathophysiologic changes observed in the lungs of patients with ARDS include exudative inflammation accompanied by diffuse damage of the alveolar–capillary membrane and accumulation of high protein edema in the alveoli [3], leading to an increase in lung tissue density causing alveolar collapse in dependent lung regions [4, 5].

Computed tomography (CT) scan is the gold standard to qualitatively detect pulmonary edema [6, 7]. However, quantification of pulmonary edema using chest CT scan has never been part of routine diagnostics due to its complexity and time-consuming nature [8]. Therefore, in recent years, measuring extravascular lung water (EVLW) with single-indicator transpulmonary thermodilution (TPTD) has become increasingly popular to detect and quantify pulmonary edema at the bedside [9–11].

Artificial intelligence algorithms (AI) can be used to segment lung CT images automatically within a relatively short time [12]. Nevertheless, there is a paucity of data validating the quantification of pulmonary edema using lung segmentation on CT compared with TPTD [13].

Therefore, the primary endpoint of the present study was to validate the quantification of pulmonary edema using automated lung segmentation on CT compared with TPTD in patients with moderate-to-severe ARDS. As secondary endpoints, we performed manual segmentation of the CT scans and compared the calculated pulmonary edema to the results retrieved from automated lung segmentation. In addition, we performed a local voxel density analysis to quantify the effects of contrast agents on our results.

## Methods

### Ethical approval

This retrospective, observational, single-center case–control study was approved by the local ethics committee (Medizinische Ethikkommission II, University Medical Centre Mannheim, Medical Faculty Mannheim of the University of Heidelberg, Mannheim, Germany; registration number 2021–831) and registered at the German Clinical Trials Register (DRKS00026115). This study was conducted at the 25-bed ICU in the Department of Anesthesiology and Critical Care Medicine, University Hospital Mannheim.

A detailed description and workflow of the institutional management of patients with moderate-to-severe ARDS, as well as TPTD, can be found in the Additional Files.

### Inclusion and exclusion criteria, data acquisition

This study analyzed all patients with moderate-to-severe ARDS (quotient between the arterial partial pressure of oxygen and the fraction of inspired oxygen < 150 mmHg at a PEEP level of at least 5 cm H2O) admitted to the ICU between January 2016 and December 2021, who had a chest CT scan with simultaneous hemodynamic monitoring using TPTD. ARDS was diagnosed according to current definitions [14, 15].

Due to the interference of extracorporeal membrane oxygenation (ECMO) with the measurement of the extravascular lung water (EVLW) by TPTD, patients on ECMO support were excluded from the study [16]. We did not exclude CT scans with contrast agent [17]. Furthermore, we did not exclude patients with pleural effusion or pneumothorax. If there were multiple CT scans from the same patient, only the first was considered for the purpose of the study to avoid repeated measurements. Anthropometric data and clinical characteristics were acquired from a retrospective review of electronic medical records (Philips Intelli Space Critical Care and Anaesthesia) at the date of admission of the patients on the ICU. Physiological data and TPTD measurements were extracted from the system at the nearest possible timepoint to the CT scan.

### Computed tomography scan

Images of the lungs were acquired using a second-generation dual-source CT scanner (Somatom Definition Flash) with 32 × 0.6 mm collimation, 89/76 reference mAs at 120 kV, a pitch of 0.8, and 0.5 s rotation time. A slice thickness of 1.5 mm, increment of 1.2 mm and medium–soft convolution kernels (I31f; Q33f) were chosen for all CT scans. Contrast agent was used according to the attending radiologist. According to the standard operating procedure of our institution, in patients with moderate-to-severe ARDS, chest CT images were acquired at a timepoint indicated by the attending physician.

### Quantification of pulmonary edema using automated lung segmentation on CT

We conducted a retrospective quantitative analysis of chest CT scans utilizing 3DSlicer (http://www.slicer.org) [17], specifically employing the Lung CT Segmenter tool in conjunction with the R-231 model from the Chest Imaging Platform for automated segmentation [18]. Voxel density was expressed as Hounsfield unit (HU). Lung weight was calculated as previously described by Protti et al. [18]:

$$\text{Voxel tissue weight = }\left(\text{1 }- \, \left(\text{Voxel density / }-{1000}\right)\right)\times \text{ Voxel volume}$$

$$\text{Calculated lung weight = (1 }-\text{ (Mean HU / }-\text{1000)) }\times \text{Lung volume}$$

The values for $\text{Mean HU}$ and lung volume were measured [19] and the expected lung weight was calculated as described by Cressoni et al. [20]:

$${\text{Expected}}\,{\text{lung}}\,{\text{weight}} = - 1806.1 + 1633.7 \times {\text{Patient's}}\,{\text{height}}\,{\text{(m)}}$$

Assuming that 1 g of lung fluid is equivalent to 1 ml of edema, the pulmonary edema derived from automated segmentation (PEauto) was calculated as follows:

$$\text{PEauto (ml) = Excessive lung weight = Calculated lung weight }-\text{ Expected lung weight}$$

Furthermore, we refined the automated analysis by including only voxels with Hounsfield Units (HUs) associated with pulmonary edema (− 700 to 200 HU) [22, 23]. We also excluded voxels with HUs associated with contrast agents (> 200 HU) [24, 25]. The corrected pulmonary edema (PEautocorr) was then calculated analogue to the method described above for automatic segmentation.

The accuracy of the automated lung segmentation was post hoc analyzed and graded into three groups (1–3) according to the segmentation quality using a three-point semiquantitative scoring system (see also the Additional Files). In addition, we performed a manual segmentation (segment editor from 3DSlicer) of all CT scans analyzed for the calculation of pulmonary edema (PEmanual) using the segment editor from 3DSlicer. This manual segmentation, conducted by an intensive care physician, was used to further evaluate the quality of the automated segmentation.

### Quantification of pulmonary edema using TPTD

We conducted a retrospective review of electronic medical records and TPTD measurements using Philips Intelli Space Critical Care and Anesthesia (ICCA). Pulmonary edema measured via TPTD (PETPTD) was calculated assuming that all extravascular lung water index (EVLWI) values > 7 ml/kg are pathologic and correspond to the amount of pulmonary edema [21]. The physiologic fraction of lung water was, therefore, calculated as

$$\text{Physiological lung water }(\text{ml)}\text{ = 7 ml/kg }\times \, {\text{IBW}}$$

where IBW is the ideal body weight. Accordingly, pulmonary edema was calculated as

$${\text{PE}}_{\text{TPTD}}\text{=}{\text{ EVLWI}}_{\text{TPTD}} - \, {\text{Physiological lung water}}_{\text{TPTD}}\text{=}{\text{ EVLWI}}_{\text{TPTD}} - \, \text{(7 ml/kg }\times \text{ IBW)}$$

As TPTD measurements were not recorded contemporaneously to the CT scan, we allowed a 24-h synchronization window for comparative edema quantification with both methods as rapid changes in EVLWI are unlikely [22–24].

### Statistical analysis

The number of patients was calculated based on a preliminary data from a previous study conducted by our group [25] in which we found a difference in the quantification between pulmonary edema with CT and TPTD of 30 ml and a standard deviation of 290 ml and a maximum difference of 700 ml. Therefore, calculating with a sample size of 145 patients resulted in a power higher than 80% for Bland–Altman analysis. We only included one comparative measurement from each patient in the analysis. Anthropometric characteristics as well as CT and TPTD findings were analyzed using a two-sample *t* test and Mann–Whitney U test, respectively. Normally distributed continuous variables are presented using means ± standard deviation, non-normally distributed continuous variables are presented using median (25% and 75% quartile) values. Nominal data were analyzed using Fisher's exact test.

Agreement between PETPTD, PEauto, PEautocorr and PEmanual was tested first using Spearman´s correlation. Furthermore, a Bland–Altman plot was used to visualize the agreement between PETPTD, PEauto, PEautocorr and PEmanual [26]. The data are expressed as mean between both measurement modalities. Bias was calculated as the mean of the differences of both measurement modalities. Ninety-five percent limits of agreement were calculated as the mean difference ± 1.96 times the standard deviation of the differences. We calculated the Jaccard Index and DICE similarity coefficient as previously described [27]. Statistical significance was set at *p* < 0.05. Statistical analysis was performed using IBM SPSS Statistics 25. All figures were created using GraphPad Prism Version 10.

## Results

Between January 2016 and December 2021, we identified 145 mechanically ventilated patients with moderate-to-severe ARDS at admission to the ICU with simultaneous measurement of lung edema using chest CT and TPTD.

The anthropometric and relevant clinical characteristics of the study population on the day of the CT scan are presented in Table 1. The average time between ARDS onset and the CT scan was 3.5 ± 4.6 days. The average time between the CT scan and the TPTD measurement was 4.8 ± 5.4 h (Table 1).

**Table 1:** Anthropometric and clinical characteristics of the study population on the day of the CT scan

	All (*n* = 145)
Age (years)	60 ± 15
Weight (kg)	90 ± 26
Height (cm)	173 ± 10
Body mass index (kg/m^2^)	30 ± 8
Female (%)	33%
SAPS II score at admission	61 ± 14
ICU length of stay (days)	17 (8–26)
Time between ARDS onset and CT scan (days)	3.5 ± 4.6
Time between CT scan and TPTD measurements (hours)	4.8 ± 5.4
Hemodialysis/CRRT (%)	19%
Fluid balance from admission to time of CT (ml)	0 (− 284 to 410)

Anthropometric and clinical characteristics parameters of 145 patients with ARDS. Data are presented as means ± standard deviation or median (Q1–Q3)

CT: computed tomography; SAPS: Simplified Acute Physiology Score; ICU: intensive care unit; CRRT: continuous renal replacement therapy

Respiratory settings, hemodynamic measurements, and laboratory data acquired immediately before or after the CT scan are shown in the Additional Files (Table S1, Table S2, and Table S3, respectively). There were no systematic changes in the ventilator settings between the CT scan and the TPTD measurements. PEEP was set to 13 ± 4 cm H2O and did not differ at the time of the CT and the TPTD measurement.

Table 2 shows air and tissue distribution in the lung parenchyma based on analysis of the chest CT scans. The calculated lung weight of the automated segmentation was 1681 ± 507 g and the expected lung weight was 1019 ± 161 g, giving a calculated excessive lung weight of 66 ± 47%.

**Table 2:** Air and tissue distribution in the lung parenchyma based on chest CT scan analysis

Parameter	Automated segmentation	Manual segmentation	Automated Corrected segmentation	*p* value
Weight				
Expected lung weight (g)	1019 ± 161	1019 ± 161	1019 ± 161	
Calculated lung weight (g)	1681 ± 507	1712 ± 578	1657 ± 504	0.806
Excessive lung weight (ml)	661 ± 472	692 ± 546	637 ± 473	0.769
Excessive lung weight* (%)	66 ± 47	69 ± 54	64 ± 47	0.759
Anatomy				
Lung volume (ml)	3545 ± 1199	3614 ± 1241	3613 ± 1237	0.961
Aerated lung volume (ml)	1952 ± 1090	1901 ± 1134	1956 ± 1089	0.830
Lung tissue volume (ml)	1681 ± 507	1712 ± 578	1657 ± 504	0.806

Air and tissue distribution of 145 patients with ARDS calculated either from automated or manual segmentation of the chest CT scans. Automated corrected segmentation only considers Hounsfield units associated with pulmonary edema (− 700 to 200 HU). Data is presented as means ± standard deviation and analyzed with a Student *t* test or the Mann*–*Whitney *U* test as appropriate

CT: computed tomography

^*^Excessive lung weight (%) = [(Calculated lung weight−Expected lung weight)/Expected lung weight] *100

**Fig. 1:**
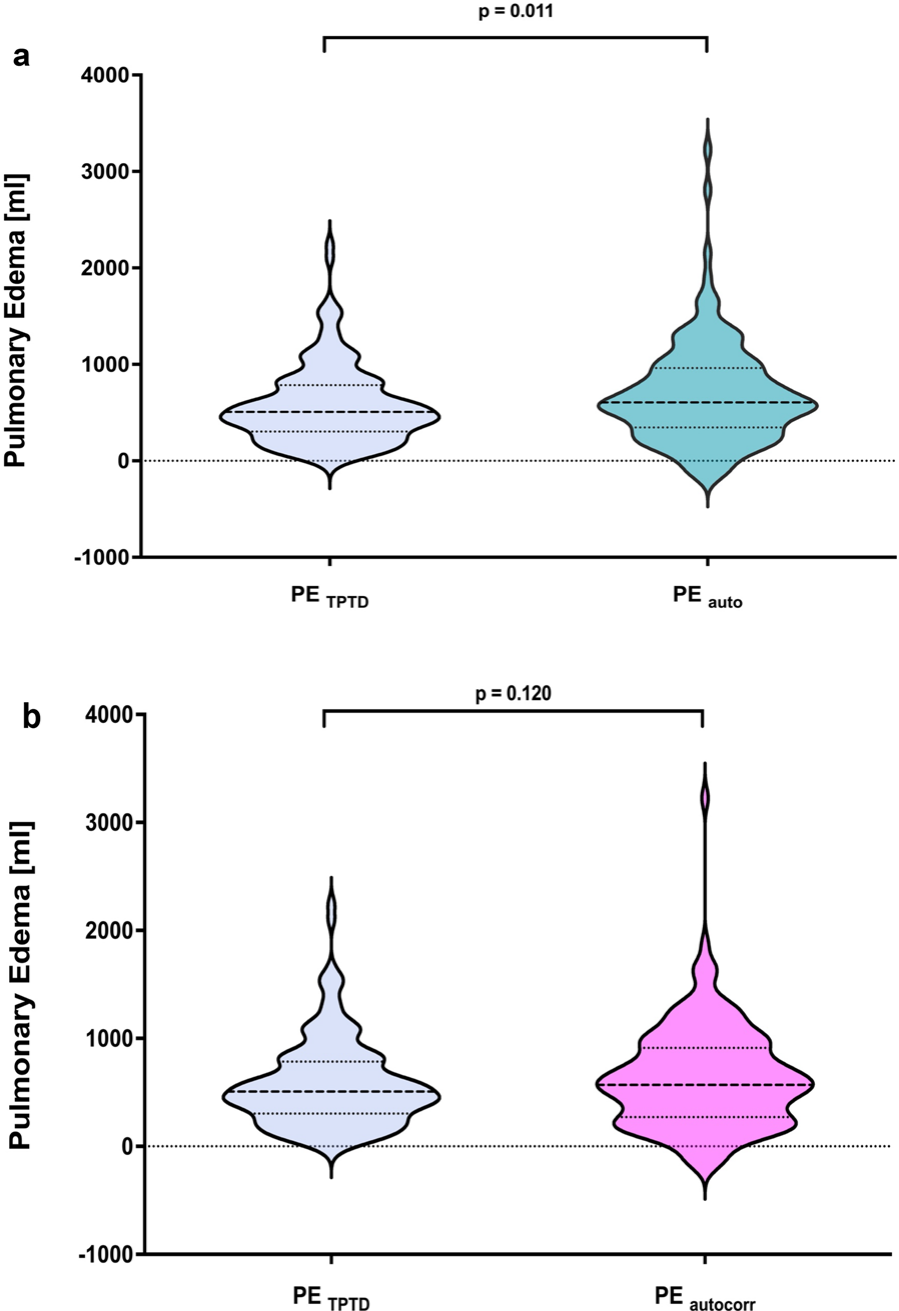
Pulmonary edema in patients with moderate-to-severe ARDS. Violin plots comparing pulmonary edema quantified using TPTD (blue) to **a** automated segmented CT scans automated (turquoise) and to **b** automated segmented CT scans using local voxel density analysis and excluding contrast agent (magenta). Data distribution is represented by the violin plot with solid and dotted lines showing the median and interquartile range, respectively. Brackets denote statistical analysis between measurement modalities. Statistical analysis was performed using Student’s *t* test. CT: computed tomography; PE: pulmonary edema; TPTD: transpulmonary thermodilution

**Fig. 2:**
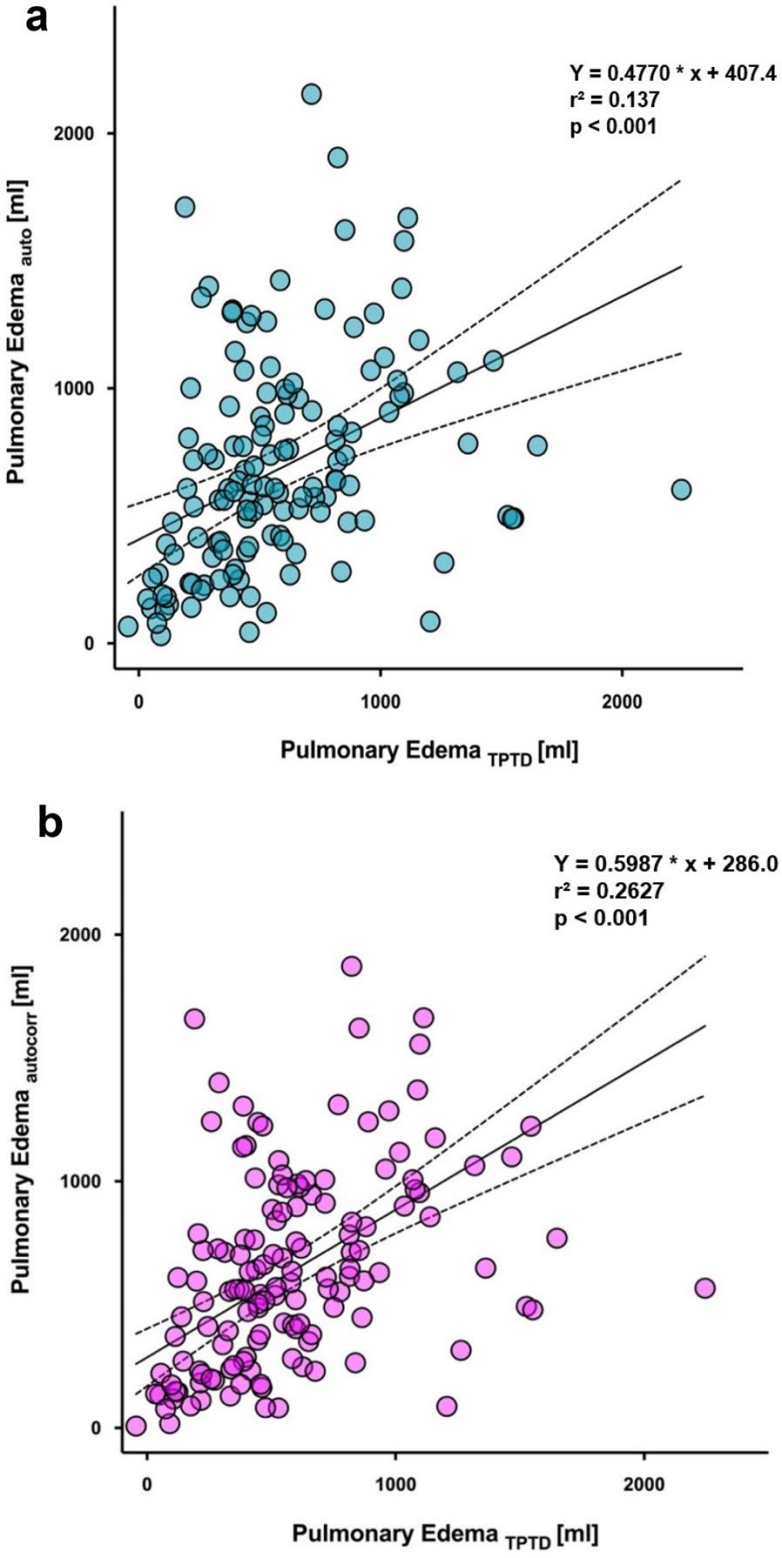
Correlation of pulmonary edema quantified using TPTD and measured by CT scan segmented **a** automated (blue circles) and **b** automated using local voxel density analysis and excluding contrast agent (magenta circles). The solid line represents the mean, while the dashed lines indicate the 95% confidence interval. Statistical analysis was performed using Spearman Correlation. CT: computed tomography; TPTD: transpulmonary thermodilution

PETPTD was significantly lower than PEauto [508 (305–814) ml vs 606 (347–962) ml, *p* = 0.011] (Fig. 1a), while PEautocorr did not differ from PETPTD [570 (270–912) ml vs 508 (305–814) ml, *p* = 0.120] (Fig. 1b). PETPTD correlated significantly with PEauto (*r* = 0.44, *p* < 0.001) (Fig. 2a) and PEautocorr (*r* = 0.54, *p* < 0.001) (Fig. 2b).

**Fig. 3:**
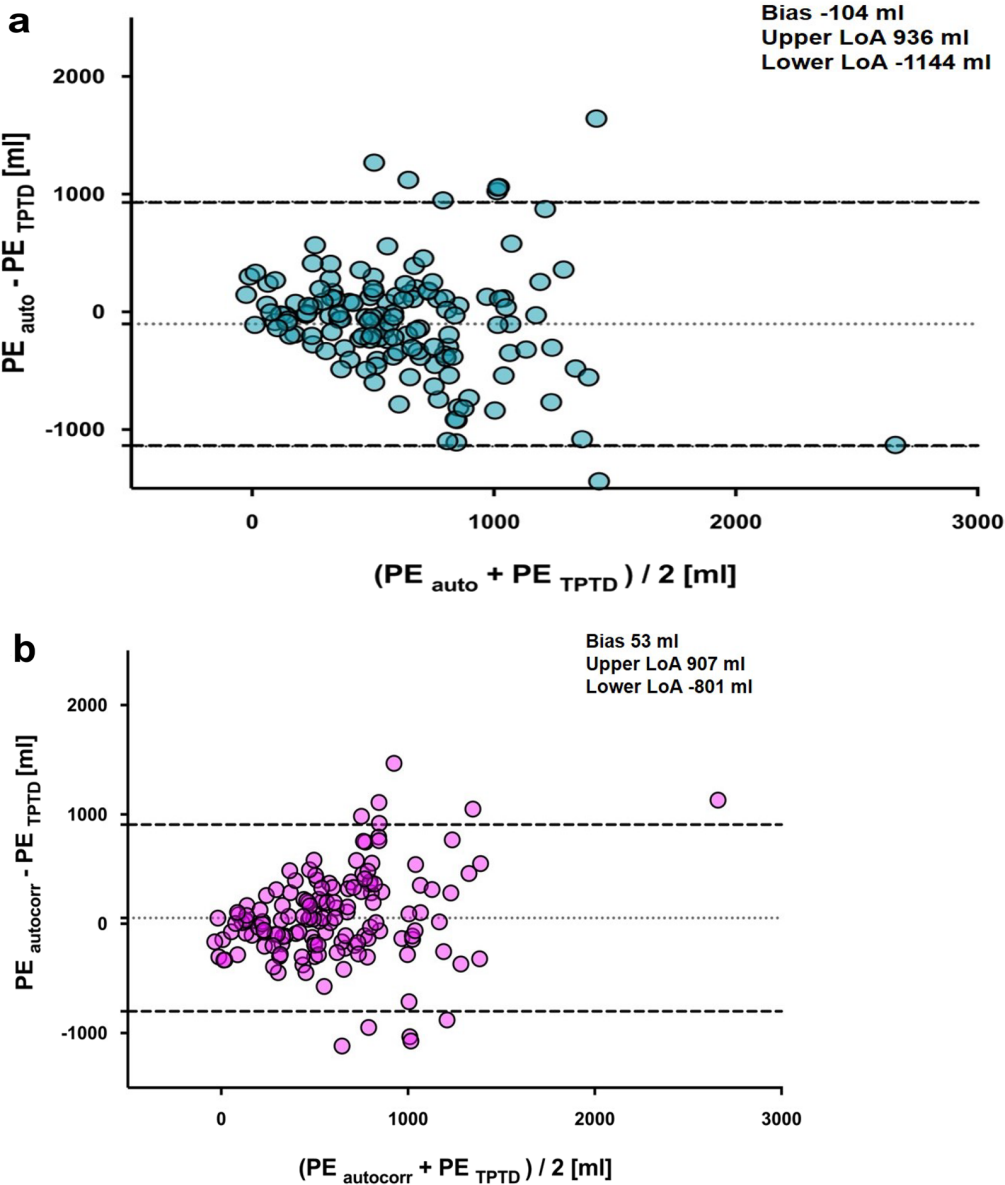
Bland–Altman plot of pulmonary edema measured by **a** automated segmentation (blue circles) and **b** automated segmentation using local voxel density analysis and excluding contrast agent (magenta circles) compared with transpulmonary thermodilution. Data are shown as the mean between pulmonary edema measured by CT scan and transpulmonary thermodilution plotted against the difference of both measurements. Dotted lines show bias, dashed lines the upper and lower levels of agreement. CT: Computed tomography; LoA: Limit of agreements; TPTD: transpulmonary thermodilution

Comparing PETPTD with PEauto using Bland–Altman analysis showed a bias of − 104 ml with a lower level of agreement of − 1144 ml and an upper level of agreement of 936 ml (Fig. 3a). Comparing PETPTD with PEautocorr showed a bias of 53 ml, a lower level of agreement of − 801 ml and an upper level of agreement of 907 ml (Fig. 3b).

The evaluation of the quality and the precision of the automated lung segmentation was graded as 1 (good) (97 patients, 67%), 2 (moderate) (29 patients, 20%) and 3 (poor) (19 patients, 13%). The Jaccard Index and DICE similarity coefficient for PEmanual with PEauto were calculated as 0.945 and 0.975, respectively. For PEmanual with PEautocorr, the Jaccard Index and DICE similarity coefficient were 0.83 and 0.88, respectively.

To further describe the quality of the automated segmentation, we manually segmented all chest CT scans analyzed in the study and calculated PEmanual. PEmanual did not differ from PEauto [609 (340–916) ml vs 606 (347–962) ml, *p* = 0.326] (Figure S3) or PE_autocorr_ (609 (340–916) ml vs 570 (270–912) ml, *p* = 0.078) (Figure S4).

Comparing with PE_TPTD_, PE_manual_ was significantly higher [508 (305–814) ml vs 609 (340–916) ml, *p* = 0.016] (Figure S5). There also was no statistically significant difference between PE_auto_ and PE_autocorr_ (606 (347–962) ml vs 609 (340–916) ml, *p* = 0.054) (Figure S6).

Furthermore, PE_manual_ correlated significantly with PE_auto_ (*r* = 0.74, *p* < 0.001), PE_autocorr_ (*r* = 0.85, *p* < 0.001) and PE_TPTD_ (*r* = 0.49, *p* < 0.001).

Furthermore, Bland–Altman analyses are provided in the Additional Files.

## Discussion

In this retrospective, observational, single-center, case–control study, the quantification of pulmonary edema was validated using automated lung segmentation on CT scans compared to single-indicator TPTD in mechanically ventilated patients with moderate-to-severe ARDS. The agreement between quantification of edema with CT and TPTD showed a low bias with a wide level of agreement and a statistically significant correlation between both measurement techniques. Manual segmentation did not differ from automated lung segmentation, whether or not corrections for contrast agents or voxel density were applied.

In moderate-to-severe patients with ARDS, the quantification of pulmonary edema in CT scans provides information of density distribution of the lung, which may influence therapeutic decisions of the attending physician [11]. The technique for lung weight measurement by chest CT imaging was established several years ago but is, when performed manually, a time-consuming process and has never been part of routine clinical diagnostics [8]. Moreover, there is a paucity of data able to estimate pulmonary edema in chest CT scans.

At the bedside, TPTD has become a well-validated measurement technique to estimate the amount of excess fluid in the lungs [28–30] with low intra- and interobserver variability [11, 31]. However, TPTD does not provide information on the anatomical distribution of edema and might be difficult to interpret in patients managed with ECMO [16] or with a significant intracardiac shunt [32].

The evolution of AI algorithms enables automated lung segmentation on chest CT images of patients with ARDS [33]. In theory, AI-supported automated lung segmentation is feasible for untrained staff at the bedside and intensive care physicians could extract clinically relevant quantitative data from CT scans within a few minutes. Visualization of changes in lung pathology and individual edema distribution might be useful for PEEP titration, the application of recruitment maneuvers [5, 34–37] or prone positioning [38] and can lead to a more personalized therapy [34–37, 39].

In a small cohort, Zhang et al. [13] demonstrated that the quantification of pulmonary edema on manually segmented CT scans using a self-designed software showed good agreement with single-indicator TPTD [13]. At first glance, our findings are in contrast to this data as we found AI-supported automated lung segmentation results in an unbiased but imprecise measurement. Zhang et al. reported a bias of − 277 ml, which is considerably higher than the bias we found [13]. Moreover, Zhang et al. did not report any limits of agreement and only showed a relatively broad 95% confidence limit, which was in the range between 200 ml and − 700 ml [13]. Nevertheless, the correlation coefficient calculated by Zhang et al. was notably higher than in this study. However, we included 15 times the number of patients in our analysis and the correlation coefficient is known to be statistically susceptible to larger sample sizes. The correlation coefficient we reported was still within a reasonable range of coefficients in studies conducted for the clinical validation of TPTD. Katzenelson et al. reported a correlation coefficient of 0.967 between transpulmonary thermodilution and gravimetric measurements in an animal model enrolling 15 dogs [29], and Kirov et al. found a coefficient of 0.85 using the same technique investigating 18 sheep [29]. Tagami et al. [40] compared pre-mortem EVLW values by single TPTD and post-mortem lung weight in 30 human patients and found a coefficient of 0.904. Venkateswaran et al. [30] found a correlation coefficient of 0.7 when comparing in vivo thermodilution EVLWI and gravimetric ex vivo EVLWI in 60 donor lungs rejected for transplant. The correlation coefficient here also decreases with the increasing number of cases.

The wide limits of agreement we found might be explained by the segmentation algorithm itself. The CT scans were graded according to the automated segmentation quality. The bias between the two measurement methods increased as the grading declined. This suggests a systematic error caused by segmentation quality. There are relatively few direct comparisons of automated segmentation algorithms analyzing chest CT scans of the whole lung with inhomogeneous parenchyma [41, 42] typically for ARDS. The combination of different algorithms might improve the performance of automated lung segmentation in the future. However, the limits of agreement between the three resulting grades when compared to TPTD remained almost constant. Therefore, the quality of the grading does not appear to be a major factor influencing the differences between the two measurement methods. This is supported by the results of the manual CT scan segmentation we conducted to rule out any systemic errors induced by the AI algorithm. Compared to TPTD, manual segmentation showed a significant difference in absolute edema quantification, just like automatically segmentation did. There also was no relevant improvement on the levels of agreements. We did not exclude CT scans acquired with contrast agents, which can influence the results of computed tomographic measurements of lung volumes in patients with acute lung injury [17]. For this reason, we performed an additional edema quantification based on local voxel density analysis. HU range was defined from − 700 [49] to 200 [50], as we considered values above this limit representing contrast agent. Even without contrast agents, wide levels of agreement were still evident. Now, however, the calculated absolute edema did not differ significantly when compared to TPTD, indicating that the use of contrast medium at least seems to have an influence on edema quantification.

Based on our data, we are unable to exclude a potential inadequacy in the quantification of pulmonary edema with TPTD due to an increase of cellular or plasmatic components in the pulmonary vasculature. Patroniti et al. reported that TPTD underestimates the amount of pulmonary edema in edematous lungs compared to manual segmentation [51]. However, we noted no statistical difference after we performed manual segmentation and compared the edema to results retrieved from TPTD (see Figure S9 in the Additional Files). Quantifying pulmonary edema with TPTD in ARDS patients correlates with the amount of parenchymal inflammation [52, 53]. It is unclear whether the pathophysiologic changes in the pulmonary vasculature, such as early vasodilation and clot formation, influence the results of EVLWI measurements by TPTD.

On the other hand, the pathophysiological changes in ARDS lungs described above might also affect CT analysis. We used established formulas to quantify pulmonary edema using CT scan data [8]. Methodologically, this approach does not account for the pooling of inflammatory cells or the shift of blood from the systemic to the pulmonary circulation. These factors could contribute to the inaccuracies in the measurement of pulmonary edema in CT scans.

In addition, ventilator settings, particularly the applied PEEP, can significantly impact EVLWI measurements with TPTD [54] and the amount of atelectasis [55], which might influence the calculation of pulmonary edema on CT. However, we observed no significant changes in PEEP between the time of the CT scan and the TPTD measurements. Therefore, we assume that the level of PEEP is of secondary importance in this analysis.

### Clinical implications

Chest CT scans provide valuable qualitative information regarding lung parenchyma inhomogeneity and density distribution for the clinician managing ARDS patients [7]. However, our data suggests that quantitatively estimating pulmonary edema using chest CT scans segmented by an AI supported algorithm results in unbiased but imprecise measurements compared to EVLWI obtained by TPTD. Although the significant correlation between the two measurement methods indicates that they are related, there may be other factors not revealed by our data, such as pulmonary hyperemia, parenchymal fibrosis, or changes in pulmonary vasculature, which could affect TPTD measurements. These factors might also contribute to weight gain in inflamed lungs. Therefore, longitudinal monitoring of pulmonary edema using CT imaging, complemented by ongoing EVLWI measurements, is recommended to capture a more comprehensive picture over time. Even though the Bland–Altman analysis presented wide levels of agreement, this analysis was hampered either by the performance of the AI algorithm [42], by the properties of the CT scan data [17] or by the fundamentally different techniques to quantify pulmonary edema.

### Limitations

This study has several limitations. Due to the retrospective nature of the study, we did not standardize the time interval between ICU admission and the chest CT scan. The time delay between TPTD measurement and the CT scan might account for the imprecision of the quantification of pulmonary edema in the CT due to volume shifts. However, providing profound differences in fluid balance on a daily base, changes in EVLWI measurement typically manifest over days [22–24].

We defined lung edema as excessive lung weight [43]. Using the formula of Cressoni et al., we followed an empirical approach to estimate the expected lung weight based on body size [20]. However, the empirical determined approximation proposed by Cressoni et al. has a coefficient of agreement of only 0.49 [20]. Despite this, the model is frequently employed in respiratory physiology [44–48]. The poor coefficient might be partly explained by the large sample size and the associated susceptibility to outliers. However, the use of an empirical model to estimate the expected lung weight was necessary as we had no preliminary CT findings of the corresponding healthy lung to calculate the actual physiological lung weight of the patient using CT segmentation. In our work, however, we aimed to quantify the pulmonary edema in particular, which has never been attempted in this form before, and not lung tissue volume using local voxel density analysis and extravascular lung water, which is considered as edema surrogate in comparable studies [21, 22]. The empirical approach we used, therefore, might create a systemic bias, which could be responsible for the broad limits of agreement we found.

Furthermore, pulmonary edema measured using TPTD was defined as the difference between EVLWI and physiological lung water. Physiological lung water was considered as up to $\text{7 ml/kg }\times \text{ IBW}$, whereas the actual range is given as ${4}-\text{7 ml/kg }\times \text{ IBW}$. Therefore, we did not consider a patient-specific, physiological lung water, which may have led to a systematic error in the current analysis.

We only included patients with moderate-to-severe ARDS in this study. This might constitute a selection bias describing a population with unique pathophysiologic properties.

## Conclusions

Automated lung segmentation on CT is an unbiased but imprecise measurement of pulmonary edema in mechanically ventilated patients with moderate-to-severe ARDS.

## Supplementary Information

Supplementary Material 1.

## Data Availability

The datasets used and/or analyzed during the current study are available from the corresponding author on reasonable request.

## Abbreviations

AI: Artificial intelligence
ARDS: Acute respiratory distress syndrome
CO: Cardiac output
CT: Computed tomography
EVLW: Extravascular lung water
EVLWI: Extravascular lung water index
HU: Hounsfield units
IBW: Ideal body weight
PEEP: Positive end-expiratory pressure
PE_auto_: Pulmonary edema automatic segmentation
PE_autocorrect_: Pulmonary edema – 700 to 200 HU
PE_manual_: Pulmonary edema manual segmentation
PE_TPTD_: Pulmonary edema transpulmonary thermodilution
SAPS II: Simplified Acute Physiology Score II
TPTD: Transpulmonary thermodilution
