# Supplementary material for: Quantification of pulmonary edema using automated lung segmentation on computed tomography in mechanically ventilated patients with acute respiratory distress syndrome
Source: Intensive Care Med Exp. 2024 Nov 2;12:95. doi: 10.1186/s40635-024-00685-w (PMC11531458; doi:10.1186/s40635-024-00685-w)
Supplement: Supplementary file 1 — Supplementary Material 1. [file 40635_2024_685_MOESM1_ESM.docx]

Quantification of pulmonary edema using automated lung segmentation on computed tomography in mechanically ventilated patients with acute respiratory distress syndrome

Alice Marguerite Conrad, Julia Zimmermann, David Mohr, Matthias F. Froelich, Alexander Hertel, Nils Rathmann, Christoph Boesing, Manfred Thiel, Stefan O. Schoenberg, Joerg Krebs, Thomas Luecke, Patricia R.M. Rocco, Matthias Otto

Additional Files

**Table of Contents**

Institutional management strategy for patients with acute respiratory distress syndrome

Figure S1 Schematic flowchart of the institutional management strategy for patients with acute respiratory distress syndrome (ARDS)

Table S1 Respiratory settings, respiratory mechanics and gas exchange of the study population on the day of the CT scan

Table S2 Hemodynamic measurements of the study population on the day of the CT scan

Table S3 Laboratory data of the study population on the day of the CT scan

Figure S2 Flow chart of the segmentation process

Grading of the automated lung segmentation

Figure S3 Comparison pulmonary edema manual *vs* automated segmentation

Figure S4 Comparison pulmonary edema manual *vs* automated, corrected segmentation

Figure S5 Comparison pulmonary edema TPTD *vs* manual segmentation

Figure S6 Comparison pulmonary edema automated segmentation *vs* automated, corrected segmentation

Figure S7 Bland-Altman plot pulmonary edema manual *vs* automated segmentation

Figure S8 Bland-Altman plot pulmonary edema manual *vs* automated, corrected segmentation

Figure S9 Bland-Altman plot pulmonary edema TPTD *vs* manual segmentation

Figure S10 Bland-Altman plot automated segmentation *vs* automated, corrected segmentation

Figure S11 Representative plots of the three segmentation grades

Figure S12 Bland-Altman plot Grade 1

Figure S13 Bland-Altman plot Grade 2

Figure S14 Bland-Altman plot Grade 3

References

# Institutional management strategy for patients with acute respiratory distress syndrome

Figure S1 shows the standard operating procedure and the management pathway of our institution for patients with acute respiratory distress syndrome (ARDS). ARDS was defined according to current definitions [1-3]. When patients with ARDS are admitted to the intensive care unit (ICU), they are managed with high-flow oxygen therapy, non-invasive or invasive ventilation as designated by the attending physician. In the case of invasive mechanical ventilation, the treatment is in accordance with the guidelines published in 2023 [4, 5]. When patients with moderate to severe ARDS (defined as a quotient between the arterial partial pressure of oxygen and the fraction of inspired oxygen < 150 mmHg at a PEEP level of at least 5 cm H_2_O) are admitted to the ICU, a chest CT scan is performed as soon as indicated by the attending physician.

Per our institutional management strategy, we standardize our ventilator management in patients with moderate to severe ARDS and use a tidal volume of 6 ml per kilogram idealized body weight [6] or a static driving pressure of less than 15 cm H_2_O [7]. We initially titrate PEEP according to the ARDS network table [6]. This may then be modified according to the attending physician. Recruitment maneuvers are not applied on a routine basis [8, 9].

All patients with moderate to severe ARDS are managed with a central venous catheter in the jugular or subclavian vein and a thermodilution catheter (Pulsiocath, Pulsion Medical Systems, Munich, Germany) in the femoral artery for the measurement of advanced hemodynamics with transpulmonary thermodilution (TPTD) [10]. As suggested by the manufacturer and following the standard operating procedure of our institution, the TPTD system is calibrated at least once every 8 h using three 20-ml injections of cold saline (4°C).


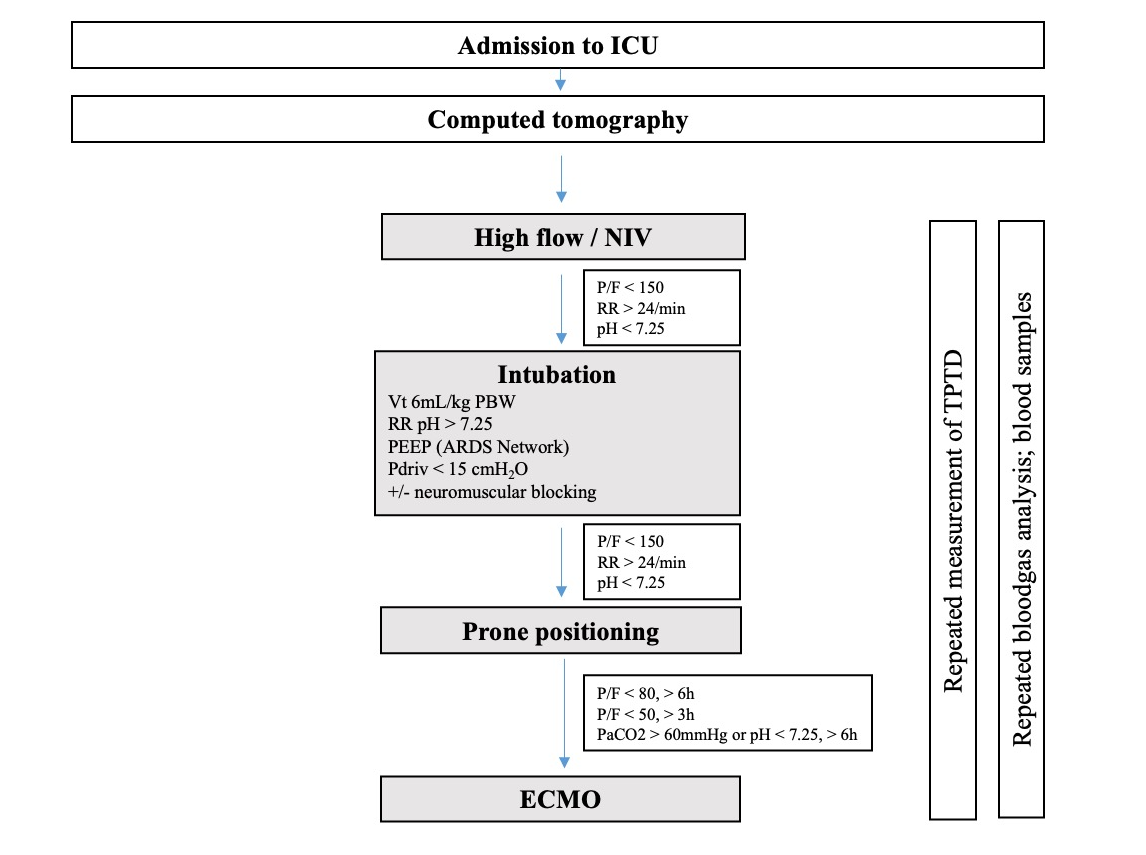


**Figure S1** Schematic flowchart of the institutional management strategy for patients with acute respiratory distress syndrome (ARDS). ECMO, extracorporeal membrane oxygenation; ICU, intensive care unit; NIV, non-invasive ventilation; PBW, predicted body weight; PaCO_2_, arterial partial pressure of carbon dioxide; PEEP, positive end-expiratory pressure; P/F, arterial PaO_2_ (“P”) divided by the FIO_2_ [(“F”) – the fraction (percent) of inspired oxygen]; Pdriv, driving pressure; RR, respiratory rate; TPTD, transpulmonary thermodilution; V_T_, tidal volume.

**Table S1** Respiratory settings, respiratory mechanics, and gas exchange for the study population on the day of the CT scan

| Parameter |  |
| --- | --- |
| PC-AC (%) | 11 |
| BIPAP (%) | 9 |
| VCV (%) | 80 |
| Pleural effusions (%) | 17 |
| Pneumothorax (%) | 5 |
| Tidal volume (ml) | 420 ± 56 |
| Respiratory rate (1/min) | 22 ± 3 |
| Peak airway pressure (cmH_2_O) | 31 ± 8 |
| Positive end-expiratory pressure (cmH_2_O) | 13 ± 4 |
| Dynamic driving pressure (cm H_2_O) | 17 ± 7 |
| Dynamic compliance of the respiratory system (ml/cmH_2_O) | 27 ± 12 |
| PaO_2_/FiO_2_ | 160 (124-217) |
| pHa | 7.3 ± 0.1 |
| PaCO_2_ (mmHg) | 53 (43-61) |
| Central venous oxygen saturation (%) | 76 ± 7 |
| Lactate (mg/dl) | 1.1 (0.8-1.6) |

Respiratory parameters of 145 patients with ARDS not caused by. Data are presented as percentage values, means ± standard deviation or median (Q1–Q3). CT, computed tomography; PC-AC, pressure-controlled assist-control; BIPAP, biphasic positive airway pressure; VCV, volume-controlled ventilation; PaO_2_/FiO_2_, ratio of arterial oxygen partial pressure to fraction of inspired oxygen; PaCO_2_, arterial partial pressure of carbon dioxide; ScvO_2_, central venous oxygen saturation; V_T_, tidal volume

**Table S2** Hemodynamic measurements of the study population immediately before CT scan

| Parameter |  |
| --- | --- |
| Heart rate (1/min) | 93 ± 21 |
| Mean arterial pressure (mmHg) | 81 ± 13 |
| Stroke volume (ml) | 74 ± 30 |
| Cardiac index (l/min/m^2^) | 3.3 ± 0.9 |
| Intrathoracic blood volume index (ml/m²) | 952 ± 287 |
| Extravascular lung water index (ml/kg) | 16 ± 6 |
| Systemic vascular resistance index (dyn*s/cm-^5^*m^2^) | 2004 ± 822 |
| Vasoactive inotropic score | 8.6 (2-21) |

Advanced hemodynamic parameters of 145 patients with ARDS. Data are presented as nominal data, means ± standard deviation, or median (Q1–Q3). CT, computed tomography

**Table S3** Laboratory data of the study population on the day of the CT scan

| Parameter | All (*n*=145) |
| --- | --- |
| Albumin (g/l) | 22 ± 5 |
| Creatinine (mg/dl) | 1.4 (0.9-2.4) |
| C-reactive protein (mg/l) | 181 (108-259) |
| White blood cells (10^9^/l) | 11 ± 6 |
| PCT (µg/l) | 1.3 (0.3-6) |
| D-Dimer (mg/l) | 3 (1-6) |

Blood measurements of patients with ARDS. Data are presented as means ± standard deviation or median (interquartile range). CT, computed tomography; PCT, procalcitonin


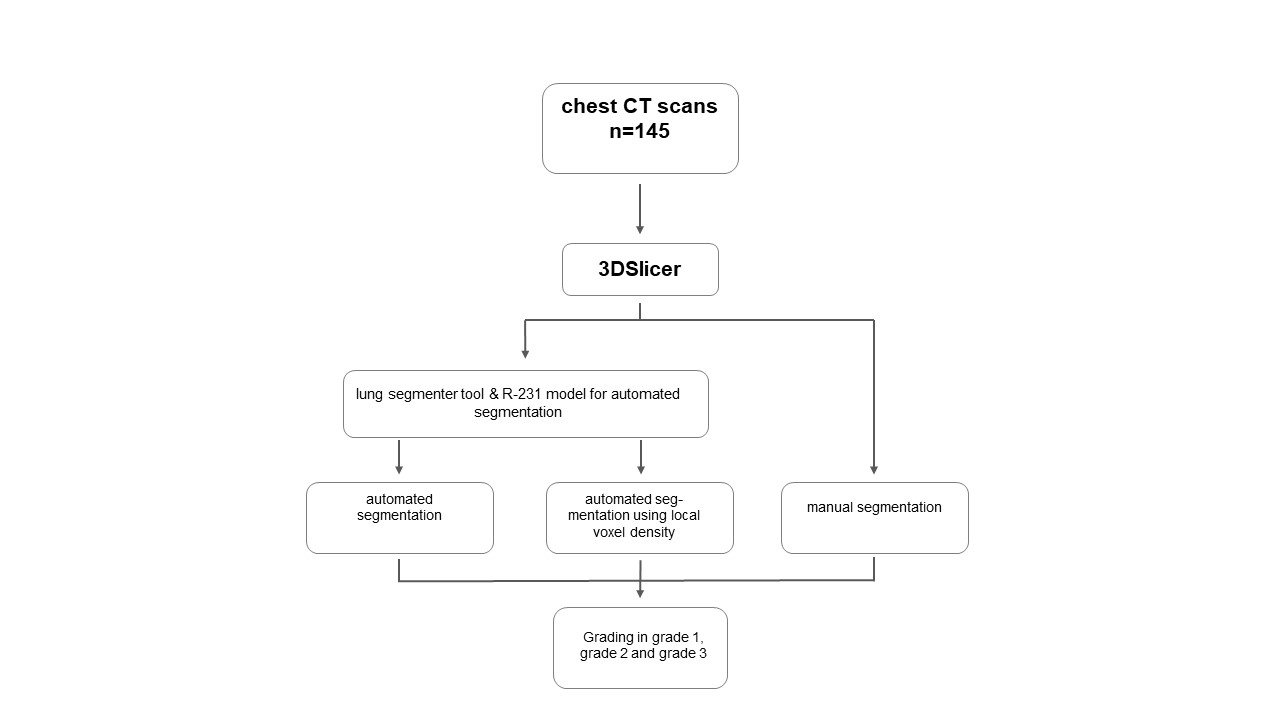
**Figure S2** Flow chart of the segmentation process. CT, computed tomograph


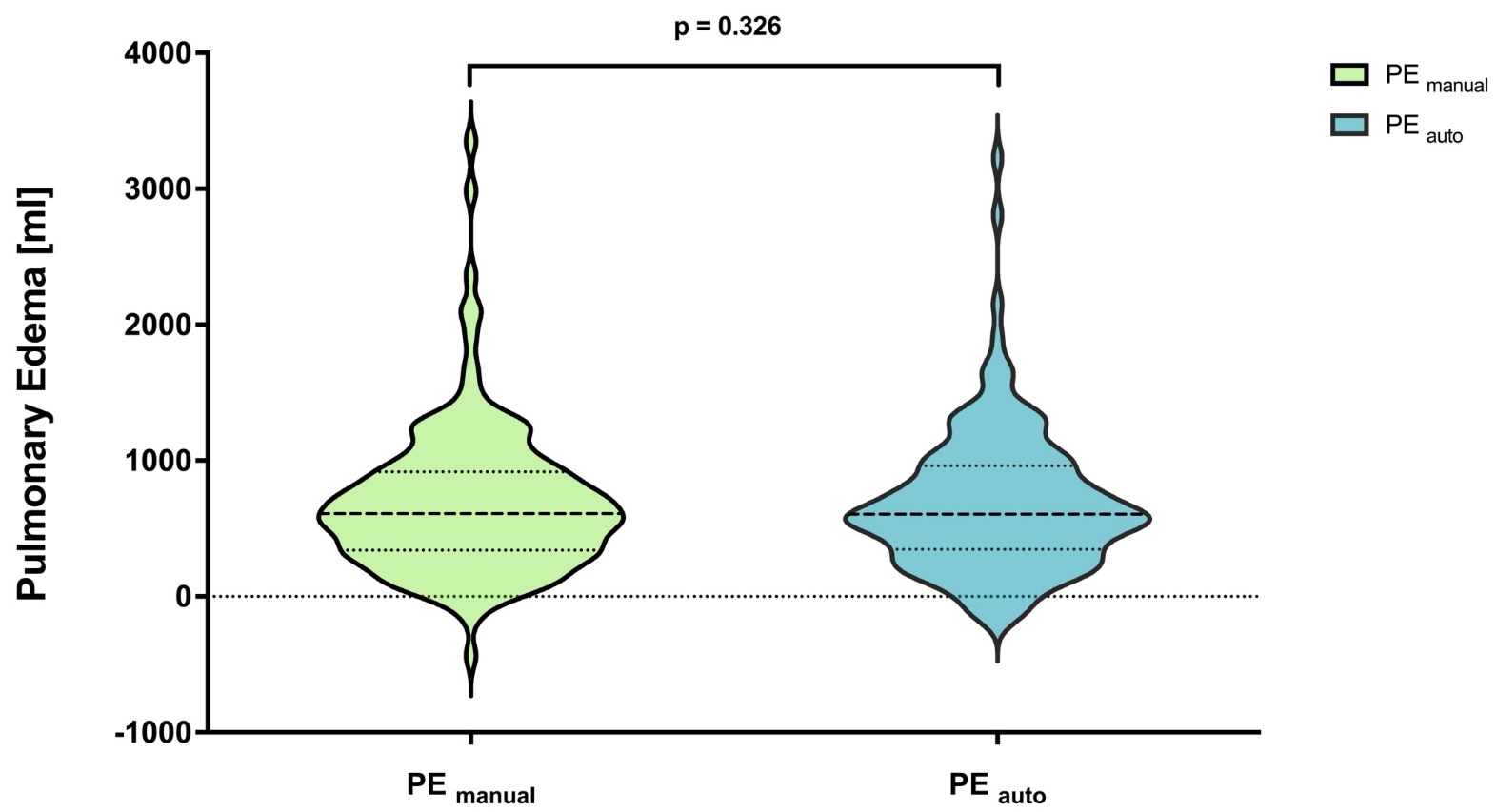


**Figure S3** Pulmonary edema in patients with moderate to severe ARDS. Violin plots comparing pulmonary edema quantified using manually (green) and automated segmented CT scans (blue). Data distribution is represented by the violin plot with solid and dotted lines showing the median and interquartile range, respectively. Brackets denote statistical analysis between measurement modalities. Statistical analysis was performed using Student's *t*-test. CT, computed tomography; PE, pulmonary edema


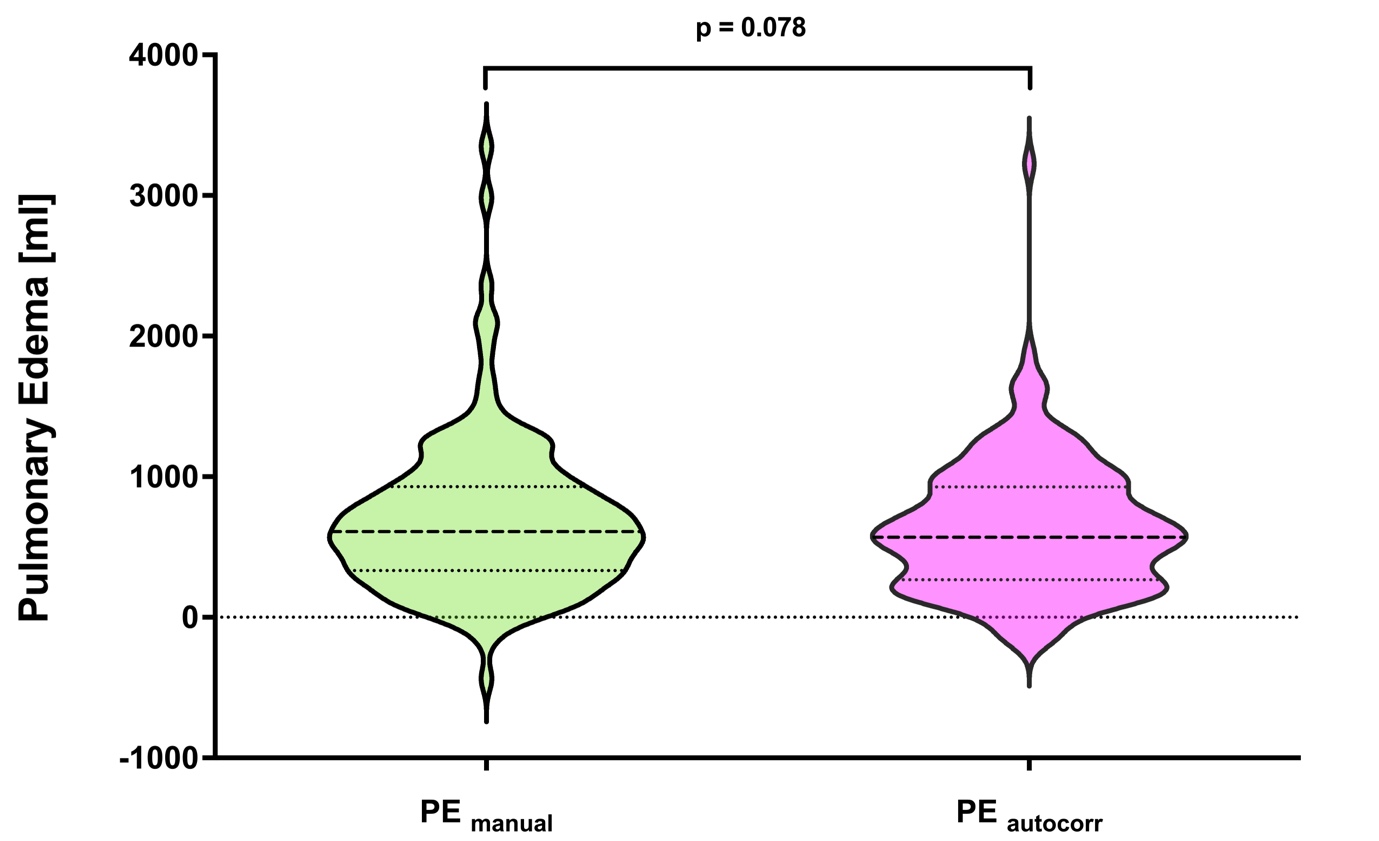


**Figure S4** Pulmonary edema in patients with moderate to severe ARDS. Violin plots comparing pulmonary edema quantified using manually (green) and automated segmented CT scans using local voxel density analysis and excluding contrast agent CT scans (blue). Data distribution is represented by the violin plot with solid and dotted lines showing the median and interquartile range, respectively. Brackets denote statistical analysis between measurement modalities. Statistical analysis was performed using Student's *t*-test. CT, computed tomography; PE, pulmonary edema


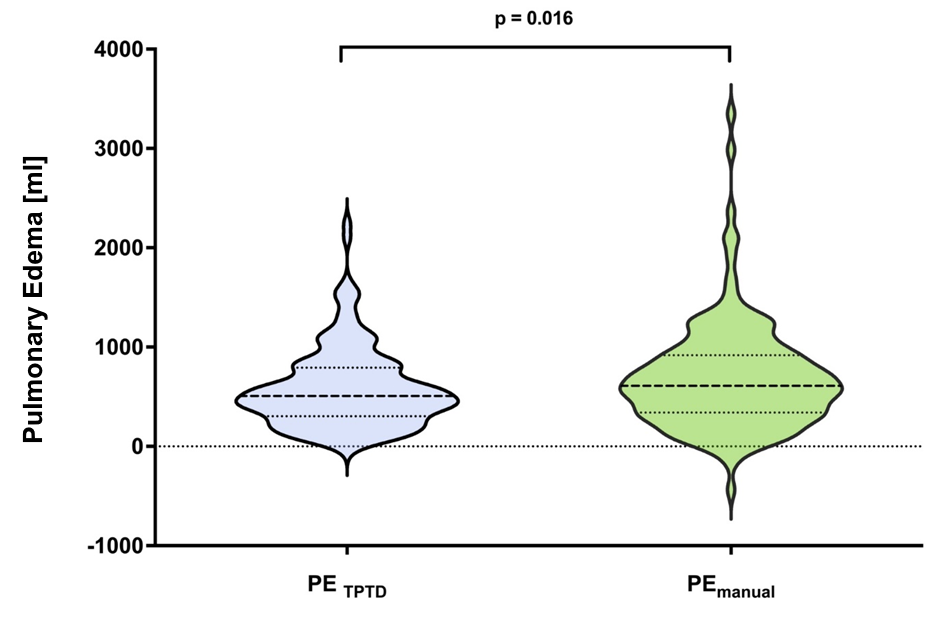


**Figure S5** Pulmonary edema in patients with moderate to severe ARDS. Violin plots comparing pulmonary edema quantified using TPTD (blue) to manually segmented CT scans (green). Data distribution is represented by the violin plot with solid and dotted lines showing the median and interquartile range, respectively. Brackets denote statistical analysis between measurement modalities. Statistical analysis was performed using Student's *t*-test. CT, computed tomography; PE, pulmonary edema; TPTD, transpulmonary thermodilution


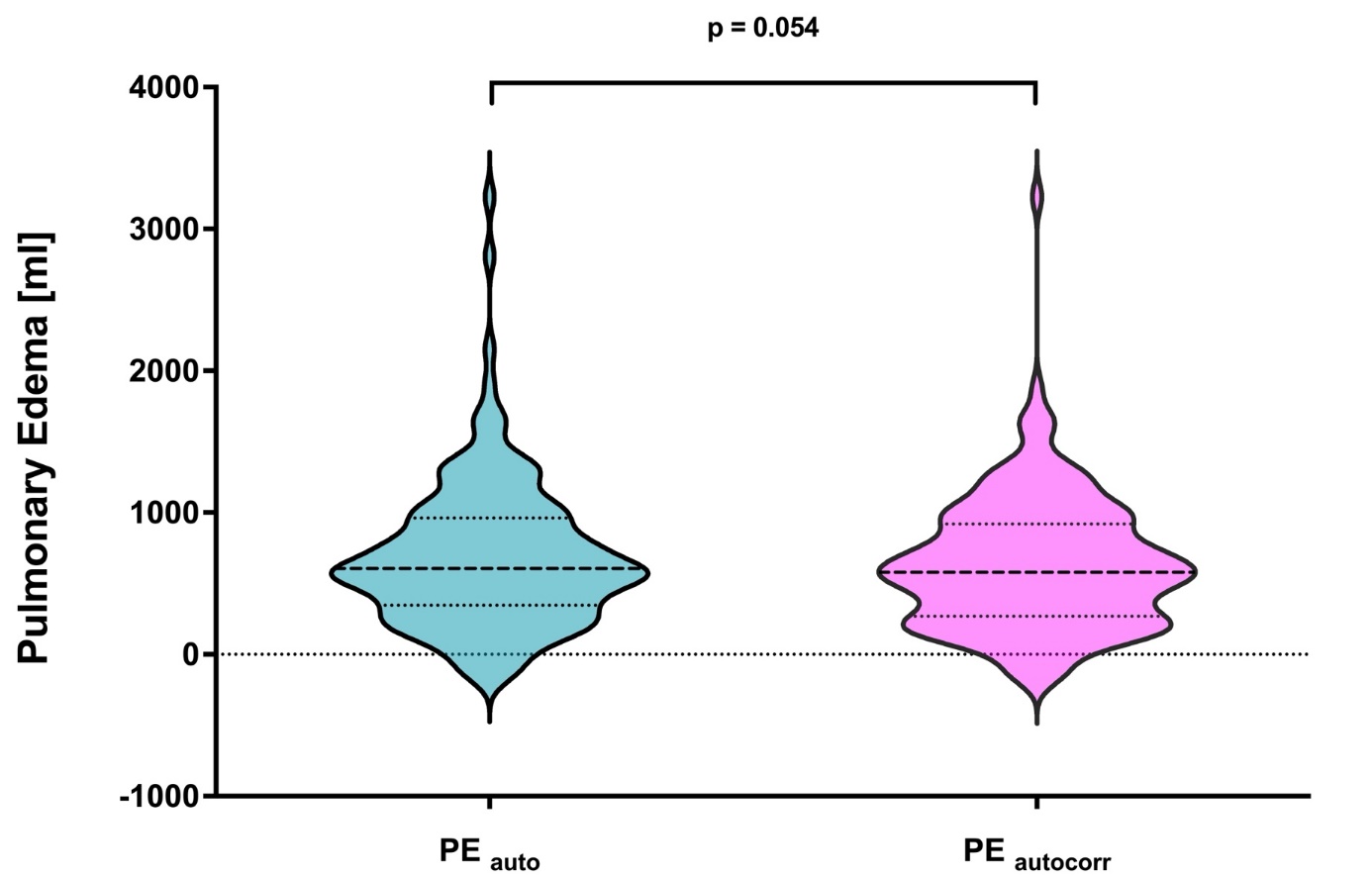


**Figure S6** Pulmonary edema in patients with moderate to severe ARDS. Violin plots comparing pulmonary edema quantified using automated (blue) and automated segmented CT scans using local voxel density analysis and excluding contrast agent (magenta). Data distribution is represented by the violin plot with solid and dotted lines showing the median and interquartile range, respectively. Brackets denote statistical analysis between measurement modalities. Statistical analysis was performed using Student's *t*-test. CT, computed tomography; PE, pulmonary edema

**
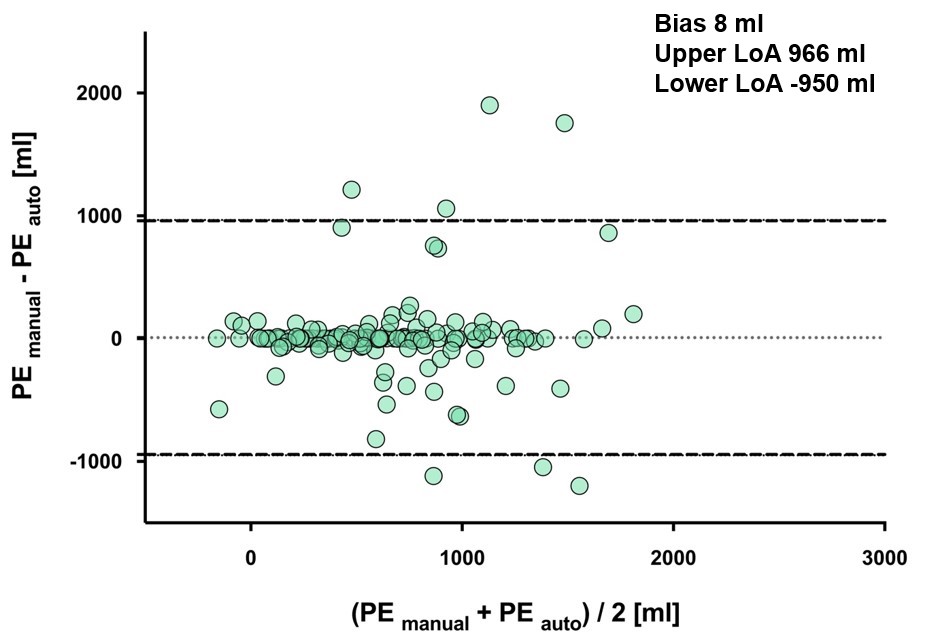
**

**Figure S7** Bland-Altman plot of pulmonary edema measured by manually segmented CT scans compared with automated segmented CT scans. Data are shown as the mean between pulmonary edema measured by manual segmentation and automated segmentation plotted against the difference of both measurements. Bias, 8 ml (dotted line); upper limit of agreement, 966 ml; lower limit of agreement, -950 ml (dashed lines)

CT, Computed tomography; TPTD, transpulmonary thermodilution; LoA, level of agreement

**
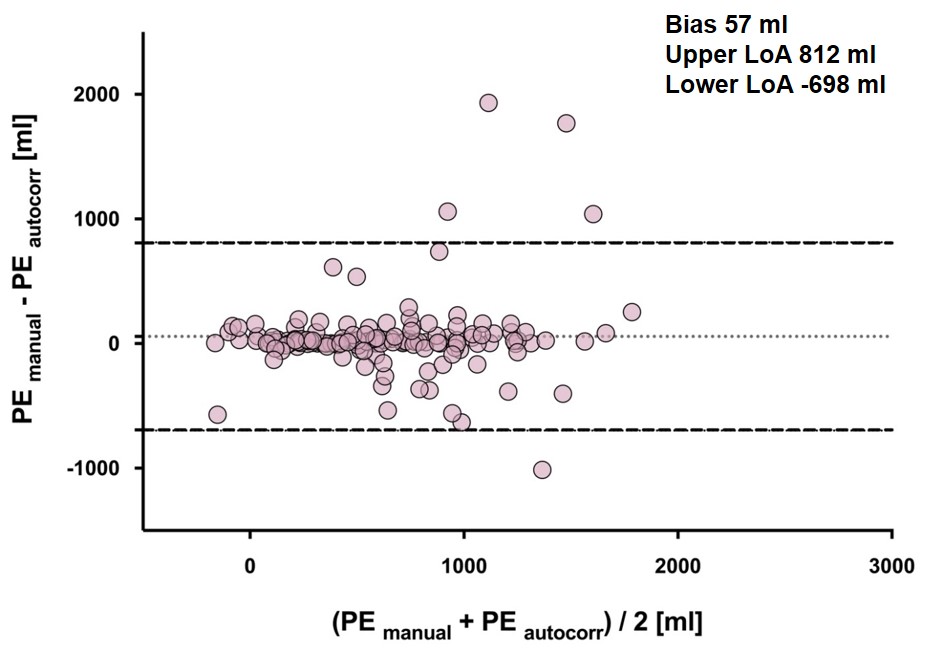
 Figure S8** Bland-Altman plot of pulmonary edema measured by manually segmented CT scans compared with automated segmented CT scans using local voxel density analysis. Data are shown as the mean between pulmonary edema measured by manual segmentation and automated segmentation plotted against the difference of both measurements. Bias, 57 ml (dotted line); upper limit of agreement, 812 ml; lower limit of agreement, -698 ml (dashed lines)

CT, Computed tomography; TPTD, transpulmonary thermodilution; LoA, level of agreement

**
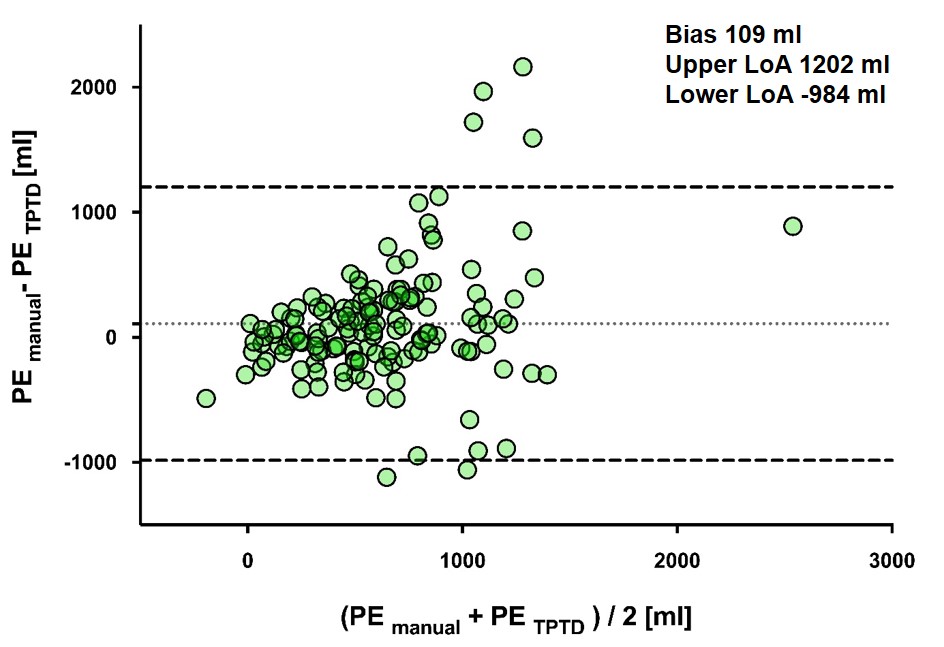
**

**Figure S9** Bland-Altman plot of pulmonary edema measured by transpulmonary thermodilution compared with manually segmented CT scans. Data are shown as the mean between pulmonary edema measured by manual segmentation and automated segmentation plotted against the difference of both measurements. Bias, 109 ml (dotted line); upper limit of agreement, 1202 ml; lower limit of agreement, -984 ml (dashed lines)

CT, Computed tomography; TPTD, transpulmonary thermodilution; LoA, level of agreement


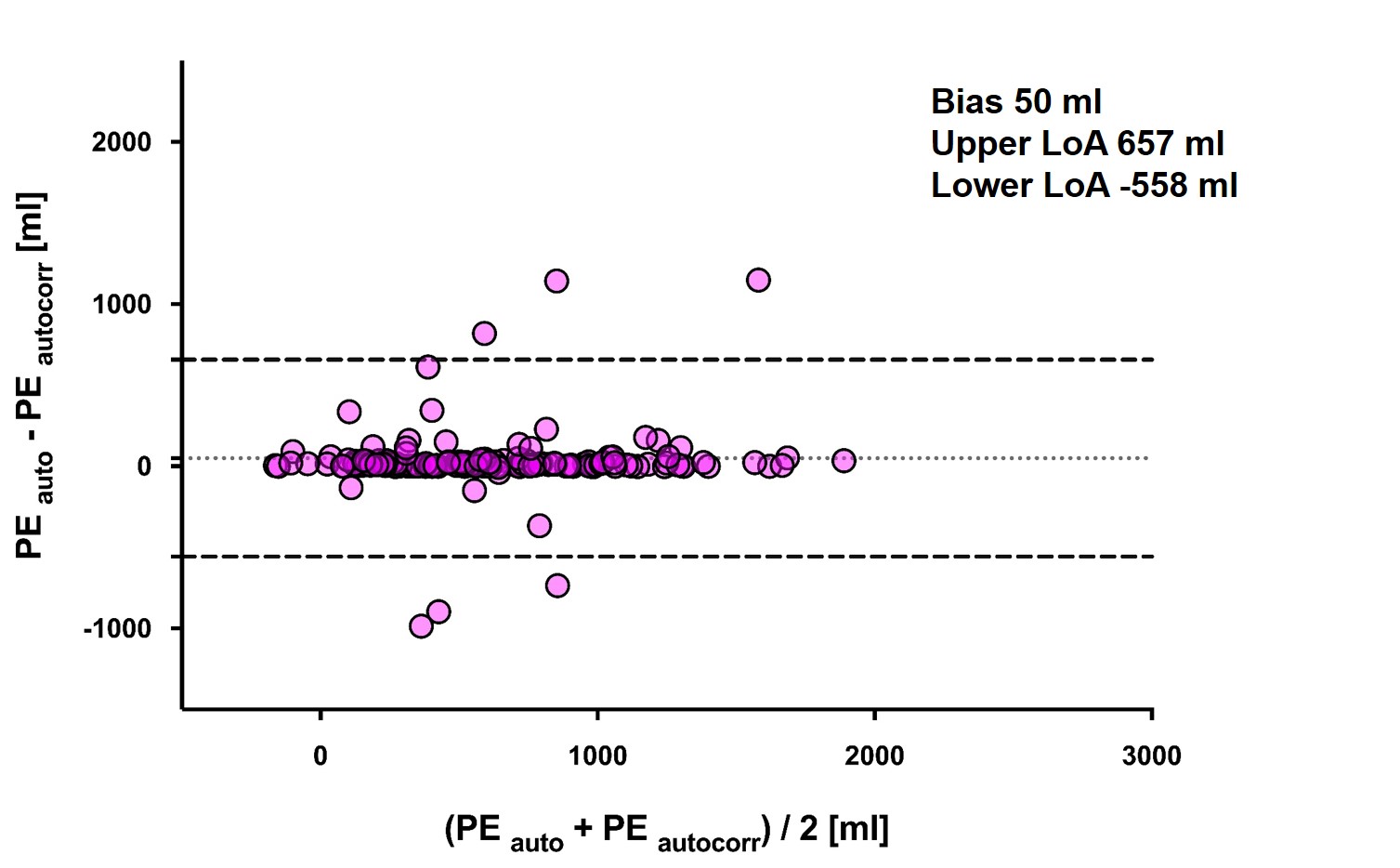


**Figure S10** Bland-Altman plot of pulmonary edema measured by automated segmented CT scans compared with automated segmented CT scans using local voxel density analysis and excluding contrast agent. Data are shown as the mean between pulmonary edema measured by manual segmentation and automated, corrected segmentation against the difference of both measurements 50 ml (dotted line); upper limit of agreement, 657 ml; lower limit of agreement, -558 ml (dashed lines)

CT, Computed tomography; TPTD, transpulmonary thermodilution; LoA, level of agreement

**Grading of automated lung segmentation**

The quality of the automated lung segmentation was evaluated by a dedicated investigator. A three-point semiquantitative scoring system was used to describe the agreement to a manual segmentation.

**Grade 1**: automated und manual segmentation are in full agreement (good)

**Grade 2**: differences < 1cm in more than on slide or differences > 1cm in a singular slide (moderate)

**Grade 3**: differences > 1 cm in more than on slide (poor)


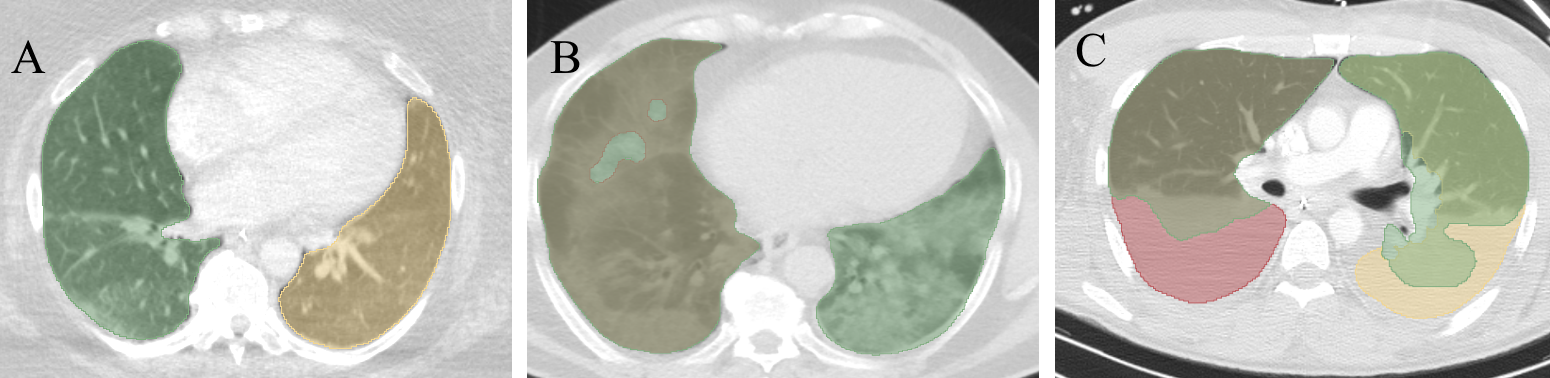


**Figure S11** Representative plots of the three segmentation grades. A: Automated segmentation Grade 1. The segmentation algorithm recognizes the lung tissue without any relevant mistakes. B: Automated segmentation Grade B. Some areas in the right lung (dark green) are not correctly recognized by the algorithm. C: Automated segmentation Grade 3: The automatic segmentation does not correctly differentiate between lung parenchyma (green) and pleural effusion (red and yellow).

**
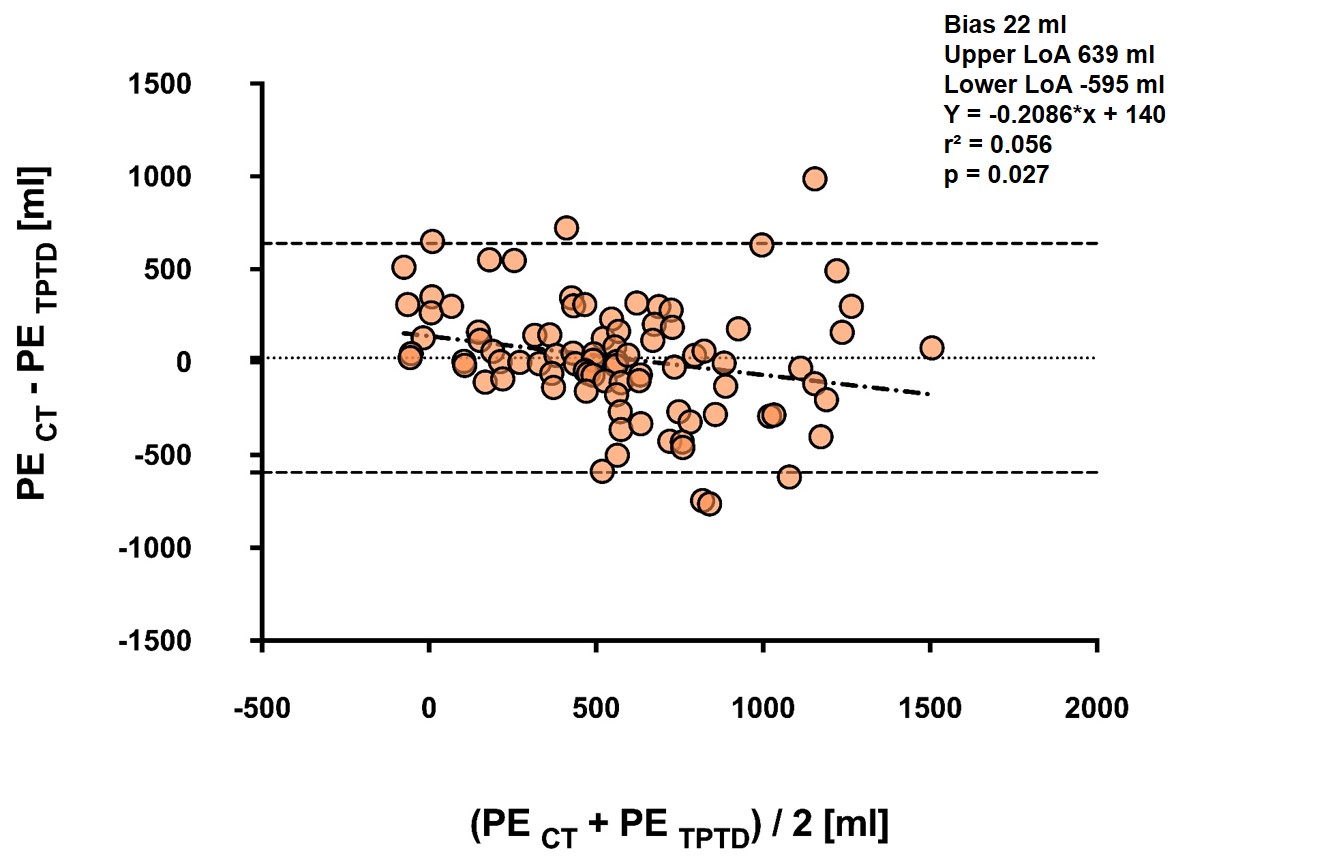
**

**Figure S12** Bland-Altman plot of pulmonary edema measured by CT scan compared with transpulmonary thermodilution in patients graded as Grade 1 by the investigator. Data are shown as the mean between pulmonary edema measured by CT scan and transpulmonary thermodilution plotted against the difference of both measurements. Bias, 22 ml (dotted line); upper limit of agreement, 639 ml; lower limit of agreement, -595 ml (dashed lines); regression, r^2^ = 0.056; p = 0.027 (segmented line). CT, Computed tomography; TPTD,

transpulmonary thermodilution; LoA, level of agreement


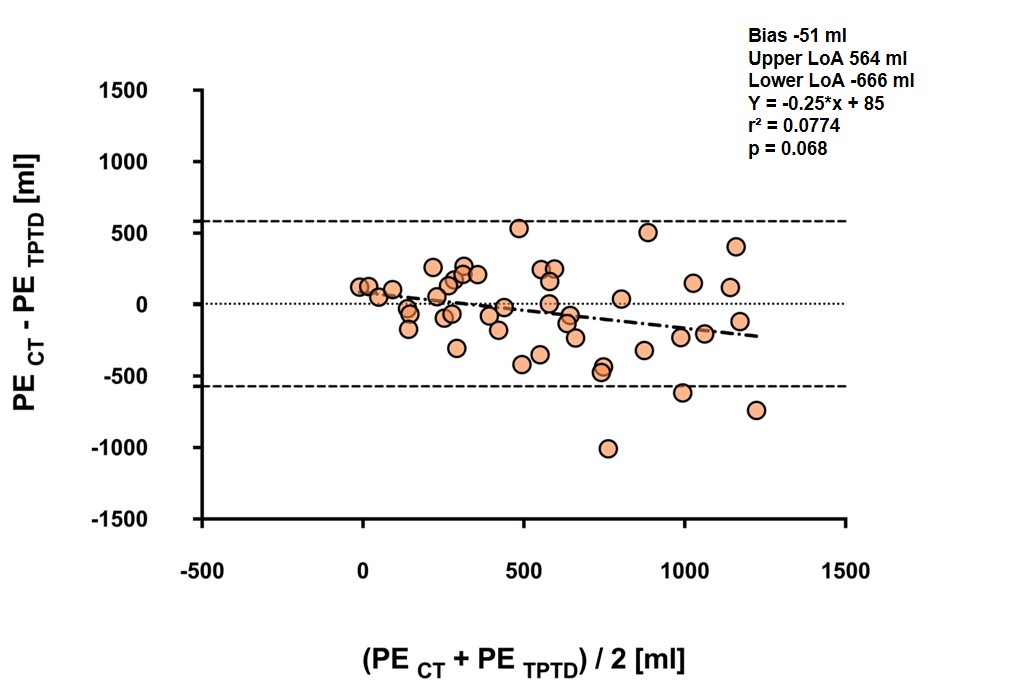


**Figure S13** Bland-Altman plot of pulmonary edema measured by CT scan compared with transpulmonary thermodilution in patients graded as Grade 2 by the investigator. Data are shown as the mean between pulmonary edema measured by CT scan and transpulmonary thermodilution plotted against the difference of both measurements. Bias, -51 ml (dotted line); upper limit of agreement, 564 ml; lower limit of agreement, -666 ml (dashed lines); regression, r^2^ = 0.0774; p = 0.068 (segmented line).

CT, Computed tomography; TPTD, transpulmonary thermodilution; LoA, level of agreement


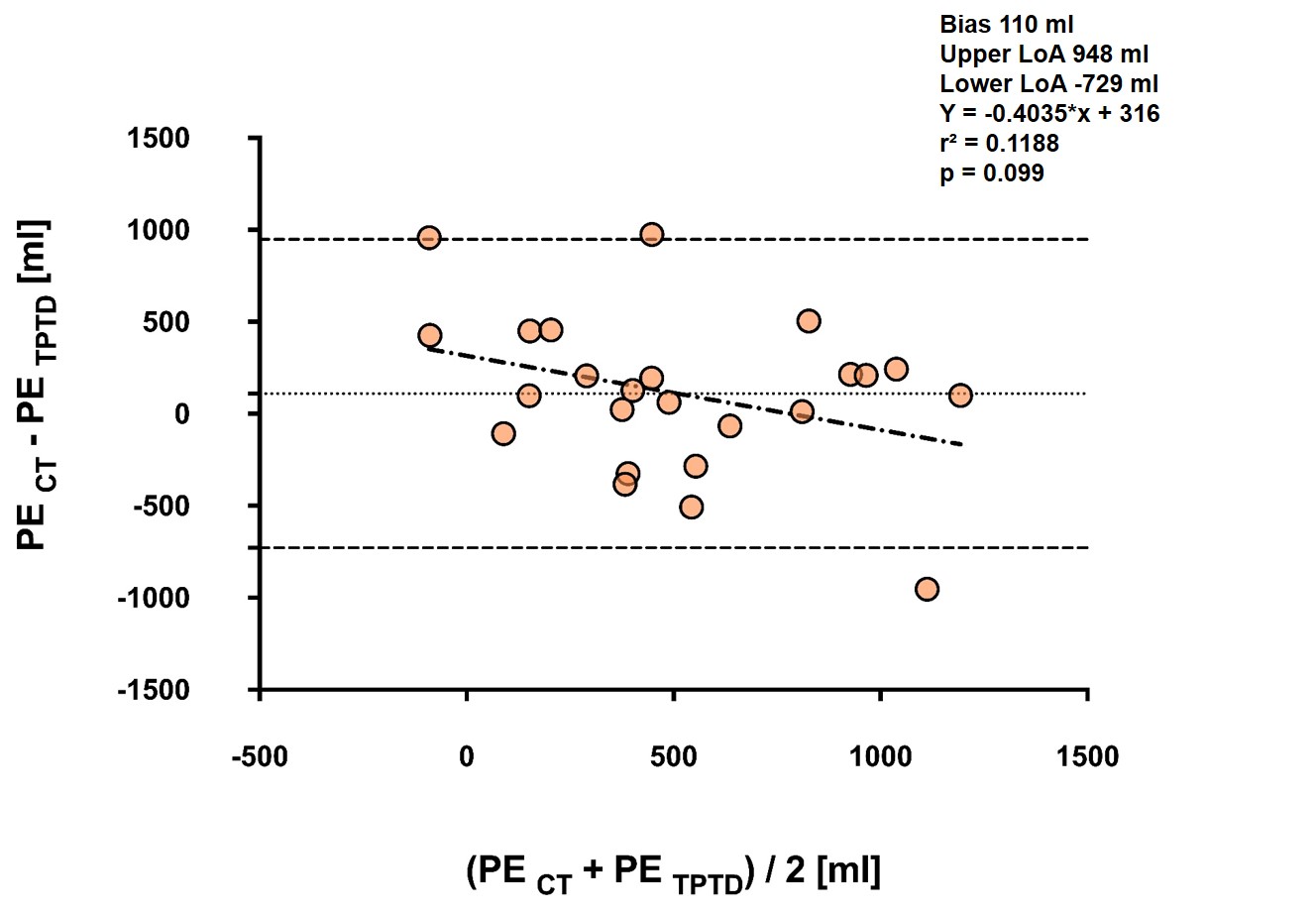


**Figure S14** Bland-Altman plot of pulmonary edema measured by CT scan compared with transpulmonary thermodilution in patients graded as Grade 3 by the investigator. Data are shown as the mean between pulmonary edema measured by CT scan and transpulmonary thermodilution plotted against the difference of both measurements. Bias, 110 ml (dotted line); upper limit of agreement, 948 ml; lower limit of agreement, **-**729 ml (dashed lines); regression, r^2^ = 0.1188; p = 0.099 (segmented line).

CT, Computed tomography; TPTD, transpulmonary thermodilution; LoA, level of agreement

References

1. Grasselli G, Calfee CS, Camporota L, Poole D, Amato MBP, Antonelli M, et al. ESICM guidelines on acute respiratory distress syndrome: definition, phenotyping and respiratory support strategies. Intensive care medicine. 2023;49(7):727-59. doi: 10.1007/s00134-023-07050-7.

2. Qadir N, Sahetya S, Munshi L, Summers C, Abrams D, Beitler J, et al. An Update on Management of Adult Patients with Acute Respiratory Distress Syndrome: An Official American Thoracic Society Clinical Practice Guideline. Am J Respir Crit Care Med. 2023. doi: 10.1164/rccm.202311-2011ST.

3. Force ADT, Ranieri VM, Rubenfeld GD, Thompson BT, Ferguson ND, Caldwell E, et al. Acute respiratory distress syndrome: the Berlin Definition. JAMA : the journal of the American Medical Association. 2012;307(23):2526-33. doi: 10.1001/jama.2012.5669.

4. Papazian L, Aubron C, Brochard L, Chiche JD, Combes A, Dreyfuss D, et al. Formal guidelines: management of acute respiratory distress syndrome. Ann Intensive Care. 2019;9(1):69. doi: 10.1186/s13613-019-0540-9.

5. Grasselli G, Calfee CS, Camporota L, Poole D, Amato MBP, Antonelli M, et al. ESICM guidelines on acute respiratory distress syndrome: definition, phenotyping and respiratory support strategies. Intensive care medicine. 2023. doi: 10.1007/s00134-023-07050-7.

6. Brower RG, Matthay MA, Morris A, Schoenfeld D, Thompson BT, Wheeler A. Ventilation with lower tidal volumes as compared with traditional tidal volumes for acute lung injury and the acute respiratory distress syndrome. N Engl J Med. 2000;342(18):1301-8. doi: 10.1056/nejm200005043421801.

7. Amato MBP, Meade MO, Slutsky AS, Brochard L, Costa ELV, Schoenfeld DA, et al. Driving Pressure and Survival in the Acute Respiratory Distress Syndrome. New England Journal of Medicine. 2015;372(8):747-55. doi: 10.1056/NEJMsa1410639.

8. Grasselli G, Calfee CS, Camporota L, Poole D, Amato MBP, Antonelli M, et al. ESICM guidelines on acute respiratory distress syndrome: definition, phenotyping and respiratory support strategies. Intensive Care Med. 2023;49(7):727-59. doi: 10.1007/s00134-023-07050-7.

9. Qadir N, Sahetya S, Munshi L, Summers C, Abrams D, Beitler J, et al. An Update on Management of Adult Patients with Acute Respiratory Distress Syndrome: An Official American Thoracic Society Clinical Practice Guideline. Am J Respir Crit Care Med. 2024;209(1):24-36. doi: 10.1164/rccm.202311-2011ST.

10. Cecconi M, De Backer D, Antonelli M, Beale R, Bakker J, Hofer C, et al. Consensus on circulatory shock and hemodynamic monitoring. Task force of the European Society of Intensive Care Medicine. Intensive Care Med. 2014;40(12):1795-815. doi: 10.1007/s00134-014-3525-z.
